# The prognostic value of baseline hematological parameters of peripheral blood in metastatic gastric cancer treated with apatinib

**DOI:** 10.7150/jca.65339

**Published:** 2022-01-01

**Authors:** Jin-Ru Yang, Dan-Yang Zhou, Ying Wu, Ying Zhu, Zhen-Yu Lin, Tao Zhang, Dan-Dan Yu

**Affiliations:** 1 Cancer Center, Union Hospital, Tongji Medical College, Huazhong University of Science and Technology, Wuhan 430022, China;; 2 Department of Medical Oncology, Sun Yat-sen University Cancer Center, State Key Laboratory of Oncology in South China, Collaborative Innovation Center for Cancer Medicine, 651 Dongfeng East Rd, Guangzhou, 510060, China.

**Keywords:** Neutrophil-to-lymphocyte ratio, CA125, albumin, apatinib, metastatic gastric cancer

## Abstract

**Background:** There is strong evidence that apatinib is effective in the treatment of third- or later-line advanced metastatic gastric cancer (mGC). Hematology prediction index is a convenient and cheap method to predict the prognosis of disease. However, the prognosis of baseline hematological parameters of peripheral blood, such as neutrophil-to-lymphocyte ratio (NLR), carbohydrate antigen 125 (CA125) and albumin (ALB) on mGC treated with apatinib have not been identified.

**Methods:** We retrospectively analyzed mGC received apatinib between 1 January 2014 and 30 June 2021. Survival analyses were performed using the Kaplan-Meier method and Cox-proportional hazards model.

**Results:** A total of 117 patients were included in this study. The cutoff value of NLR, CA125 and ALB was 2.25, 19.24 U/ml and 37.60 g/L, respectively. The disease control rates (DCR) in the high and low NLR groups were 52.94% and 73.47% (*P*=0.024); 48.28% and 74.58% (*P*=0.003) in high and low CA125 groups; 72.97% and 41.86% (*P*=0.001) in high and low ALB groups. By survival analysis, increasing NLR (*P*=0.003), CA125 (*P*<0.001) and decreasing ALB (*P*<0.001) predicted a shorter PFS after apatinib. NLR (*P*=0.015), CA125 (*P*=0.004) and ALB (*P*=0.005) were significantly predictors for PFS in mGC treated with aptinib.

**Conclusion:** Increasing NLR, CA125 and decreasing ALB were associated with poorer clinical efficiency and prognosis after apatinib treatment.

## Introduction

Tumor angiogenesis is in an un-controlled and not-organized fashion, universally considered as an essential step in tumor growth and metastasis 1. Vascular endothelial growth factor (VEGF) has been demonstrated to be the one which is majorly implicated in the pathological angiogenesis and is a signaling molecule that plays a role in several solid tumors 2. Vascular endothelial growth factor receptor 2 (VEGFR-2) is one of the most potent angiogenic factors in the VEGF-induced angiogenic signaling 3. Since gastric cancer (GC) express high VEGF and the 5-year survival of mGC with conventional chemotherapeutic agents is approximately 3.9% 4, targeting VEGF is therefore considered as an interesting therapeutic strategy for GC, especially metastatic gastric cancer (mGC). Apatinib, also known as YN968D1, inhibits VEGF-stimulated endothelial cell migration and proliferation and decreases tumor microvascular density through specifically binding VEGFR-2 5. As one of the latest orally antiangiogenic agents, apatinib makes encouraging preclinical and clinical achievements in a variety of solid tumors 6, including mGC 7. It was also approved by China Food and Drug Administration (FDA) as a subsequent-line treatment for patients with mGC in 2014. However, finding predictive biomarkers that can discriminate patients who are most likely to be sensitive to apatinib and achieve personalized therapy is one of the challenges with antiangiogenic therapies using apatinib 8.

It is reported that inflammation had been validated as a high-risk factor for several cancers 9-12. The cancer-generated inflammatory response results in upregulating cytokines and inflammatory mediators, inhibiting apoptosis, promoting angiogenesis, and damaging DNA and promoting the malignancy 9,10. Neutrophil-to-lymphocyte ratio (NLR), as a mirror of systemic inflammatory response, has been considered as a useful indicator for predicting the prognosis and survival in various cancers 11, including GC 12. Unfortunately, most of the previous studies focused on NLR as a prognostic factor in GC patients with operable early-stage 13 and metastatic disease with traditional chemotherapy 14. Relevant studies have also shown that serum indexes such as tumor markers CA125 and ALB which can reflect body nutritional status, plays an important role in diagnosis GC 15,16. However, the sensitivity of each indicator was low. It is unknown whether NLR, CA125 and ALB are suitable prognostic indicators for mGC patients receiving apatinib.

Based on this background, we aimed to evaluate whether NLR, CA125 and ALB can be used as a biomarker for predicting therapeutic response and survival outcome in mGC patients receiving apatinib.

## Methods

### Patients

We retrospectively analyzed 117 mGC patients diagnosed pathologically at cancer center, Union Hospital, Tongji Medical College, Huazhong University of Science and Technology between 1 January 2014 and 30 June 2021. Patients treated with regularly apatinib until disease progression or death in the study. Patients who had evidences of infection or hematological/autoimmune diseases, prior exposure to agents improving the number and function of blood cell, withdrawal of apatinib due to intolerable toxicity or death within 4 weeks from the first dose of apatinib therapy were excluded from the analysis. For all patients, baseline clinical-pathological characteristics, including age, gender, tumor sites, histology, Eastern Cooperative Oncology Group (ECOG) performance status, apatinib monotherapy or combination therapy and records of prior lines of chemotherapy were available for review. Baseline blood counts was defined as the results obtained within 7 days before initiating apatinib therapy. Reviewing data available at adjacent time points was to avoid selecting an outlier and ensure to record the representative outcomes. Tumor assessment was performed prior to first dose of apatinib therapy, at 2 circles of apatinib, and every 8-12 weeks thereafter, and clinical response were classified by imaging examinations of complete response (CR), partial response (PR), stable disease (SD), or progressive disease (PD) according to Response Evaluation Criteria in Solid Tumors (RECIST 1.1) 17. Degrees of therapy-related adverse events (AEs) were graded by the Common Terminology Criteria for Adverse Events (CTCAE 4.0) 18. All patients included were followed-up regularly until death or study data cutoff (30 June 2021). This retrospective study was approved by the Ethical Committees of Union Hospital, Tongji Medical College, Huazhong University of Science and Technology.

### Statistical analysis

SPSS version 22.0 (SPSS Inc., Chicago, IL, USA) was used for statistical analyses. Baseline paraments of patients included were shown using Median (Quartile) [*M* (*P_25_, P_75_*)]. NLR was defined as the absolute neutrophil count divided by the absolute number of lymphocyte count. Analysis of receiver operating characteristic (ROC) curves was used to identify the cutoff value of variables. Median value severed as the cutoff value if area under the curve (AUC) of ROC was less than 0.50. Patient characteristics was compared by Chi-squared test and Student's t-test. Progression free survival (PFS) was determined from the date of the apatinib initiation to the date of progression as evidenced by radiographic assessment or obvious clinical manifestation, or death from any cause, or last follow-up (censored). Survival analyses were conducted using the Kaplan-Meier method and Cox-proportional hazards model. All tests were performed by two-sided, and *P*<0.05 were considered statistically significant.

## Results

### Clinical characteristics

A total of 117 Asian patients receiving apatinib for mGC between 1 January 2014 and 30 June 2021 were included in the retrospective study. Due to the intolerable toxicity, the patients discontinued were excluded according to CTCAE 4.0. The baseline characteristics of 117 patients were summarized in Table 1. The median age was 53 (45-62) years old, including 45 (38.46%) females and 72 (61.54%) males. All patients received 250 mg/day of apatinib. Sixty (51.28%) patients received apatinib as a three-line or multi-line treatment. Moreover, 47 (40.17%) patients received apatinib alone and 70 patients treated with combination of other chemotherapy agents. Followed up to 30 June 2021, the median PFS after apatinib was 2.33 months.

### Baseline NLR, CA125, ALB and treatment efficacy

In the present study, the disease control rates (DCR) was 61.54% (72/117). Using ROC curves, the best cutoff for NLR, CA125, ALB were determined and the patients were divided into low NLR group (NLR<2.25) and high NLR group (NLR≥2.25); low CA125 group (CA125<3.50 U/ml) and high CA125 group (CA125≥3.50 U/ml); low ALB group (ALB<37.60 g/L) and high ALB group (ALB≥37.60 g/L); DCR in the low and high NLR groups were 52.94% and 73.47% (*P*=0.024); 48.28% and 74.58% (*P*=0.003) in high and low CA125 groups; 72.97% and 41.86% (*P*=0.001) in high and low ALB groups. No CR or PR was observed in both groups (Table 2A,B,C).

### Baseline NLR, CA125, ALB and progression-free survival

**Figure 1:**
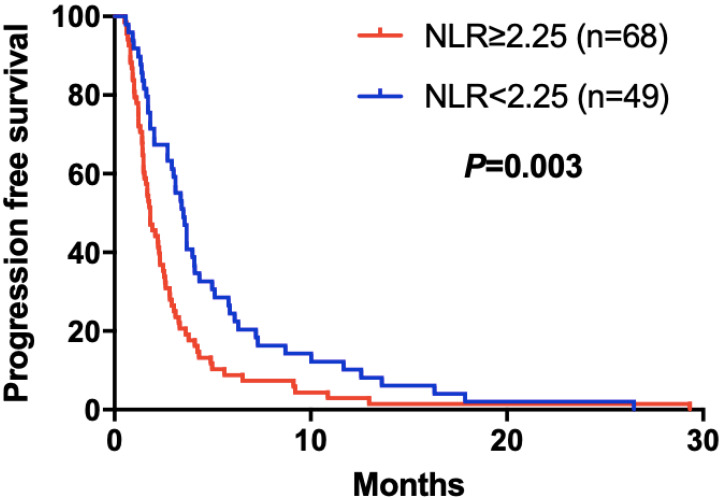
Kaplan-Meier curve for PFS of mGC patients with apatinib stratified by NLR

**Figure 2:**
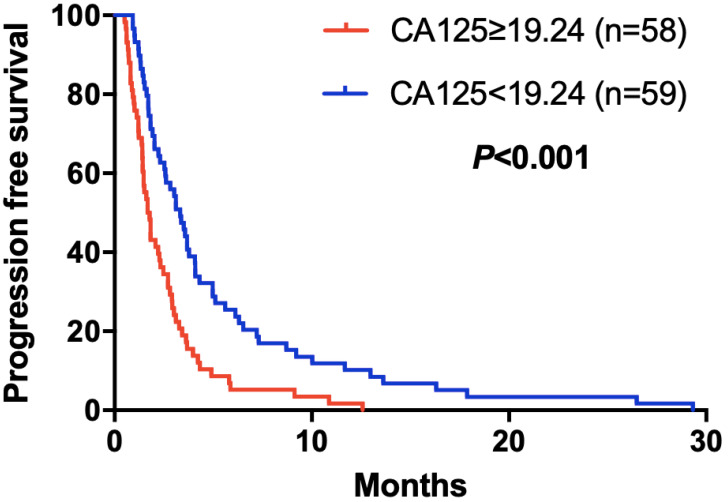
Kaplan-Meier curve for PFS of mGC patients with apatinib stratified by CA125

**Figure 3:**
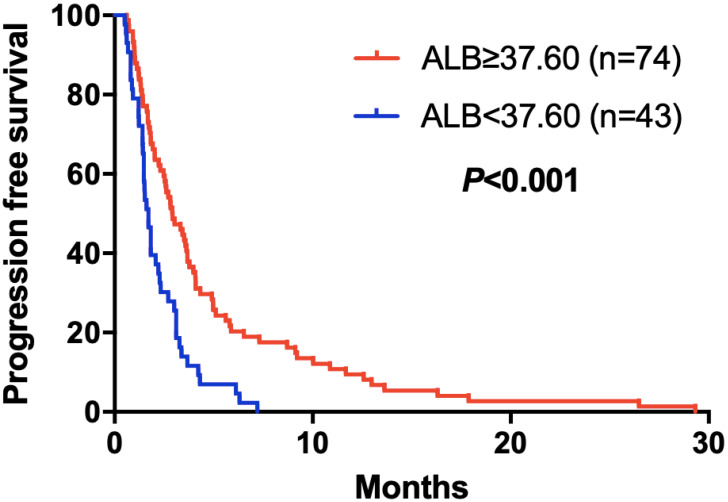
Kaplan-Meier curve for PFS of mGC patients with apatinib stratified by ALB

The elevated NLR, CA125 and reduced ALB was significantly correlated with poor prognosis for mGC patients treated with aptinib. The median PFS for patients with low NLR was 3.50 months, and for high NLR patients, it was 1.83 months (*P*=0.024); PFS was 3.33 months in low CA125 group, and 1.72 months in high CA125 group (*P*=0.003); 1.70 vs 2.92 months in low and high ALB group, respectively (*P*=0.001). (Table 2A,B,C). By Kaplan-Meier analysis, increasing NLR (*P*=0.003) and CA125 (*P*<0.001) predicted a shorter PFS after apatinib in Figure 1,2; Figure 3 showed increasing ALB (*P*<0.001) predicted a longer PFS after apatinib. Significant characteristics on univariate analysis, including gender (hazard ratio [HR]=1.496, 95% CI: 1.015-2.207, *P*=0.042), NLR (HR=0.569, 95% CI: 0.391-0.828, *P*=0.003), PLR (HR=0.576, 95% CI: 0.360-0.921, *P*=0.021), CA125 (HR=0.478, 95% CI: 0.326-0.699, *P*=0.000), ALB (HR=2.160, 95% CI: 1.446-3.225, *P*=0.000) were carried into multivariate analysis. NLR (HR=0.617, 95% CI: 0.419-0.910, *P*=0.015), CA125 (HR=0.560, 95% CI: 0.377-0.831, *P*=0.004) and ALB (HR=1.873, 95% CI: 1.214-2.892, *P*=0.005) were significantly associated with PFS by multivariate analyses (Table 3).

## Discussion

As tumor angiogenesis is one of the hallmarks of malignancy, the inhibition of VEGF signaling has become an area of considerable interest regard to anticancer therapy. Apatinib, a novel receptor tyrosine kinase inhibitor selectively targeting VEGFR-2, is considered as a promising therapeutic strategy across a range of cancers 6,19, including mGC 8,20. Compared to other anti-angiogenic agents such as bevacizumab, sunitinib and sorafenib, apatinib has demonstrated a survival advantage in GC patients 21. However, it is critically challenging to identify predictive biomarkers that could be used to select patients who will benefit most from current therapeutic strategies and avoid unnecessary cost as well as patients' discomfort.

In this study, we evaluated the prognostic value of NLR, CA125 and ALB in mGC patients with apatinib. NLR, CA125 and ALB as timesaving, inexpensive and valid prognostic factors, were independently predictive factors for mGC treated with apatinib. Elevated NLR, CA125 and reduced ALB indicated poorer clinical response and survival for these patients.

Reported previously, occurrence of adverse events might act as surrogate biomarkers of drug activity 22, such as hypertension, hand-foot syndrome, and proteinuria 23, during antiangiogenic therapy. Treatment-emergent toxic effects, as a predictor of clinical outcomes, have their own limitations in addition to exposure to useless toxicity and high cost. Many marker assessment methods have limitations when it comes to reliability and reproducibility. Hypertension is a common and serious AEs regarding to apatinib. The inconsistent result among clinical trial, the recommended threshold, the time of occurrence and withdrawal or drug reduction causing difficulties in clinical application 22. One of the advantages of baseline hematological indexes are available before the apatinib initiation compared to AEs, which can efficiently avoid the useless toxic medicine and high cost. Importantly, we further evaluate the best cutoff value of NLR (2.25), CA125 (19.24 U/ml) and ALB (37.60 g/L) in mGC treated with apatinib. High NLR, CA125 and low ALB were significantly associated with worse response to therapy and shorter PFS. Interesting, patients in the lower NLR group appeared to demonstrate superior DCR compared to previous studies 24.

High NLR means a state of neutrophilia and/or lymphocytopenia. The tumor infiltrating neutrophils (TINs), as a component of neutrophils related to tumor, produce VEGF 25, cytokines as well as cytotoxic mediators. These factors promote the epithelial-mesenchymal transition and infiltration growth of tumor cells 26 and lead to tumor proliferation, angiogenesis, invasiveness and metastasis. Neutrophil, mast cells and other immune cells are not only a source of angiogenic/lymphangiogenic molecules, but also their target 27. Neutrophils are also found to sustain tumor revascularization in the context of anti-angiogenic therapy 28. On the other hand, lymphocyte may limit tumor angiogenesis depending on the specific subtype and activation state and may induce the regression of tumor blood vessels in the context of immunotherapy 28. Apatinib as an inhibitor targeting the vascular VEGF axis may exert their activity throughout the inhibition of VEGFR-2 phosphorylation, a principal mediator in the cancer angiogenic process 29.

CA125 is a tumor associated carbohydrate antigen, and its elevated serum level is related with many malignant tumors with low sensitivity 30. Our study showed that the higher CA125 was, the shorter PFS and the lower DCR, which was consistent with the previously reported results.

Nutritional status in cancer patients is considered to be useful prognostic factors 31. Malnutrition can further cause hormone imbalance, increase inflammatory cytokine infiltration, oxidative stress and so on 32. ALB is an important serological indicator to evaluate human nutritional status. Low ALB level indicates poor nutritional status of the patient, which may lead to short PFS and DCR when treated with apatinib.

Of course, the results of this study had an inevitably deviation, because retrospective study design, and the small simple. To our knowledge, this study for the first time demonstrates a significant association between pretreatment hematological parameters of peripheral blood and apatinib therapy response in a cohort of mGC patients. This retrospective study was performed to preliminarily investigate the prognostic values of NLR, CA125 and ALB in mGC patients treated with apatinib, which is of great clinical value. Furthermore, this study provided theoretical and clinical evidence for further prospective studies.

## Conclusion

Apatinib is a promising therapeutic strategy for mGC. However, it is critically challenging to identify predictive biomarkers. Baseline hematological parameters of peripheral blood (NLR, CA125 and ALB) as simple, inexpensive and readily available biomarkers, effetely predict response to apatinib and survival.

## Figures and Tables

**Table 1:** General characteristics of mGC patients treated with apatinib (N=117)

Variable	n (%)
Age (y)	53 (45-62)
Gender	
Male	72 (61.54%)
Female	45 (38.46%)
Tumor sites	
Antrum	39 (33.33%)
Non-antrum	63 (53.85%)
Unknown	15 (12.82%)
Histology	
Poor differentiation	65 (55.56%)
Non-poor differentiation	21 (17.95%)
Unknown	31 (26.50%)
ECOG	
0-1	104 (88.89%)
2	13 (11.11%)
Apatinib alone	
Yes	47 (40.17%)
No	70 (59.83%)
Lines of treatment	
<3	57 (48.72%)
≥3	60 (51.28%)
NLR	2.64 (1.56-4.53)
PLR	170.86 (111.60-236.51)
MLR	0.36 (0.26-0.53)
CA125 (U/ml)	19.20 (10.85-49.95)
CA199 (U/ml)	17.00 (5.09-309.80)
CEA (ug/L)	5.04 (2.08-17.75)
ALB (g/L)	39.30 (36.00-41.95)
GLB (g/L)	27.30 (24.40-30.50)
ALP (U/L)	88.00 (70.00-126.50)
Progress-free survival (m)	2.33 (1.42-4.10)

**Table 2A:** Effect of baseline NLR on clinical outcomes

Clinical outcomes	All (N=117)	NLR≥2.25 (N=68)	NLR<2.25 (N=49)	*χ^2^*	*P*
Response rate					
SD	72	36	36	5.070	0.024
PD	45	32	13		
PFS (months)	2.33	1.83	3.50	-2.341	0.021

**Table 2B:** Effect of baseline CA125 on clinical outcomes

Clinical outcomes	All (N=117)	CA125≥19.24 U/ml (N=58)	CA125<19.24 U/ml (N=59)	*χ^2^*	*P*
Response rate					
SD	72	28	44	8.548	0.003
PD	45	30	15		
PFS (months)	2.33	1.72	3.33	-3.288	0.002

**Table 2C:** Effect of baseline ALB on clinical outcomes

Clinical outcomes	All (N=117)	ALB≥37.60 g/L (N=74)	ALB<37.60 g/L (N=43)	*χ^2^*	*P*
Response rate					
SD	72	54	18	11.123	0.001
PD	45	20	25		
PFS (months)	2.33	2.92	1.70	3.893	0.000

**Table 3:** Cox proportional hazard regression analysis of progression-free survival in mGC patients treated with apatinib (N=117)

Factors	Univariable		Multivariable	
	HR (95% CI)	*P*	HR (95% CI)	*P*
Age (≥ 53 vs < 53)	1.340 (0.927-1.935)	0.119		
Gender (M vs F)	1.496 (1.015-2.207)	**0.042**	1.253 (0.835-1.881)	0.276
Tumor sites (Antrum vs non-antrum)	1.303 (0.977-1.737)	0.072		
Histology (Poor vs non-poor differentiation)	1.110 (0.888-1.386)	0.360		
ECOG (0-1 vs 2)	1.124 (0.612-2.064)	0.707		
Apatinib alone (Yes vs no)	0.994 (0.682-1.448)	0.974		
Lines of treatment (≥ 3 vs < 3)	0.983 (0.678-1.426)	0.928		
NLR (≥ 2.25 vs < 2.25)	0.569 (0.391-0.828)	**0.003**	0.617 (0.419-0.910)	**0.015**
PLR (≥ 257.61 vs < 257.61)	0.576 (0.360-0.921)	**0.021**	0.787 (0.473-1.309)	0.357
MLR (≥ 0.33 vs < 0.33)	0.707 (0.484-1.031)	0.072		
CA125 (≥ 19.24 vs < 19.24 U/ml)	0.478 (0.326-0.699)	**0.000**	0.560 (0.377-0.831)	**0.004**
CA199 (≥ 9.25 vs < 9.25 U/ml)	0.794 (0.541-1.166)	0.239		
CEA (≥ 2.08 vs < 2.08 ug/L)	1.130 (0.738-1.732)	0.573		
ALB (≥ 37.60 vs < 37.60 g/L)	2.160 (1.446-3.225)	**0.000**	1.873 (1.214-2.892)	**0.005**
GLB (≥ 22.80 vs < 22.80 g/L)	0.906 (0.516-1.591)	0.732		
ALP (≥ 65.50 vs < 65.50 U/L)	0.787 (0.481-1.290)	0.342		

## Abbreviations

ECOG: Eastern Cooperative Oncology Group performance status
NLR: neutrophil-to-lymphocyte ratio
PLR: platelet-to-lymphocyte ratio
MLR: monocyte-to-lymphocyte ratio
ALB: albumin
GLB: globulin
ALP: alkaline phosphatase
PFS: progression-free survival
SD: stable disease
PD: progressive disease
